# Factors Influencing Lymph Node Positivity in HER2/neu+ Breast Cancer Patients

**DOI:** 10.3390/curroncol30030215

**Published:** 2023-02-27

**Authors:** Katherine Englander, Neha Chintapally, Julia Gallagher, Kelly Elleson, Weihong Sun, Junmin Whiting, Christine Laronga, Marie Catherine Lee

**Affiliations:** 1 University of South Florida Morsani College of Medicine, Tampa, FL 33602, USA; 2 Department of Breast Oncology, Moffitt Cancer Center, Tampa, FL 33612, USA; 3 Department of Biostatistics and Bioinformatics, Moffitt Cancer Center, Tampa, FL 33612, USA

**Keywords:** breast cancer, axillary lymph node metastasis, HER2/neu+

## Abstract

Axillary lymph node metastases are a key prognostic factor in breast cancer treatment. Our aim was to evaluate how tumor size, tumor location, and imaging results correlate to axillary lymph node diseases for patients with stage I-III HER2/neu+ breast cancer. This is a single-institution retrospective chart review of female breast cancer patients diagnosed with primary invasive Her2/neu+ breast cancer who were treated with upfront surgical resection from 2000–2021. Of 75 cases, 44/75 (58.7%) had nodal metastasis, and there was a significant association of larger tumor size to nodal metastases (*p* ≤ 0.001). Patients with negative nodes had a smaller mean tumor size (*n* = 30; 15.10 mm) than patients with positive nodes (*n* = 45; 23.9 mm) (*p* = 0.002). Preoperative imaging detected suspicious nodes in 36 patients, and ultrasound detected the most positive nodes (14/18; *p* = 0.027). Our data confirms that tumor size at diagnosis is correlated with a higher likelihood of axillary involvement in patients with Her2/neu+ breast cancer; notably, a large proportion of Her2/neu+ breast cancers have metastatic involvement of axillary lymph nodes even with small primary lesions.

## 1. Introduction

Breast cancer is the most common cancer affecting women worldwide, with an incidence in North America in 97 out of 100,000 women [1]. Mortality has decreased due to improved screening, prevention, and treatments [1]. Nodal involvement is one of the best prognostic factors in breast cancer because the lymph nodes in the axilla are often the first place that cancer cells may spread to from the breast. Many factors that influence nodal involvement have been evaluated, including tumor size, distance from the nipple, location, and receptor subtype. Tumor size has been shown to be a predictor of nodal involvement in several studies [2,3,4,5]. Smaller tumor distance to the nipple has also been reported as an independent predictor of axillary nodal involvement [5]. Studies have conflicting results on the tumor location associated with the highest likelihood of nodal disease. Both the retro-areolar and upper-outer quadrant or axillary tail have been reported to correlate with higher nodal involvement [2,4]. 

Breast cancer is divided into categories based on receptor subtype, which were initially used to guide only systematic treatment, but are increasingly being considered in surgical management as well. The receptors frequently targeted by treatment are the estrogen receptor (ER), progesterone receptor (PR), and the cell surface protein ERRB2, commonly known as HER2/neu. Breast cancers are classified into four groups by gene expression analysis, however, surrogate classification based on immunohistochemistry is an accepted clinical practice. Luminal A and Luminal B tumors are typically ER positive and PR positive (and HER2/neu negative), triple negative (ER/PR/HER2neu negative), and HER2/neu positive. HER2/neu is overexpressed in 20% of breast cancers [6]. HER2/neu is a tyrosine kinase receptor that activates genes involved in growth and differentiation of cells [6]. The overactivation of this receptor leads to increased angiogenesis, invasion, and proliferation of tumor cells [6]. Consequently, the HER2/neu+ subtype is associated with higher rates of early recurrence and metastasis [6]. Several studies have shown that HER2/neu positivity correlates with higher lymph node positivity [3,4,7]. 

The treatment and evaluation of patients with negative nodal involvement or 1–2 positive lymph nodes has evolved over recent years. Sentinel lymph node biopsy (SLNB) is often used to stage breast cancer. The prevalence of additional axillary metastasis in patients with positive sentinel lymph nodes has been reported to be 14.7–50.3% depending on the size of the metastasis in the SLN and the number of sentinel nodes with metastasis [8]. SLNB done without axillary lymph node dissection is now the standard for patients with clinically negative axillary lymph nodes (ALN), particularly those undergoing breast conserving surgery [9]. 

The necessity of SLNB in the radiologically negative axilla has been further investigated by many studies [10,11] Pooled data from four trials including 5139 patients suggests that pre-operative axillary ultrasound (AUS) has a negative predictive rate of 0.951 (95% confidence interval 0.941–0.960) [10]. It has also been suggested that patients with a radiologically positive axilla may progress straight to ALND without SLNB, but the false positive rates of imaging currently lead to 43.2% of patients undergoing unnecessary nodal clearance [12]. Because nodal burden is correlated with more advanced disease and a higher likelihood of recurrence, the accuracy of preoperative imaging must be further examined before using it to guide treatment [13].

A relationship between tumor size, location, distance to the nipple, and receptor status to nodal disease have all been established in the literature. However, these relationships have all been reported independent of hormone receptor subtype. Breast cancer is a complex and heterogenous disease, with different subtypes and molecular mechanisms. It is of interest to identify potential biological markers that can predict lymph node metastases [14]. Further evaluation of the behavior of specific subtypes of breast cancer is necessary to individualize treatment of patients. This is particularly important in the HER2/neu positive subtype, as these patients often experience earlier recurrence and higher rates of metastasis [3]. The primary aim of our study was to evaluate the relationship between tumor size and axillary lymph node disease for patients with stage I–III HER2/neu+ breast cancer. Our secondary aim was to examine how tumor location and imaging results correlate to lymph node disease in this patient population

## 2. Materials and Methods

This is a single-institution retrospective chart review of female breast cancer patients based on existing clinical data obtained from the National Comprehensive Cancer Network database, the Moffitt Cancer Center Tumor Registry, the Moffitt Historic Breast Database (MCC #16114/IRB #Pro00000091), the Breast Tumor Board Database, PowerChart, CPT codes from billing, and MCC pharmacy records. Our study draws from data submitted by the Moffitt Cancer Center to the national database from 1/1/2000 through 01/31/2021. All patients are 18 years or older and were diagnosed with primary invasive Her2/neu+ breast cancer treated with upfront surgical resection by breast surgical oncologists at a tertiary cancer center. Patients were matched by age at presentation, stage, and BMI at presentation. Multicentric, bilateral, inflammatory, and in situ-only cases were excluded. 

Medical records were reviewed in the electronic medical record (EMR), and multiple data points were collected including tumor size, tumor distance to the nipple, tumor location, imaging mode used to visualize suspicious axillary nodes, last follow up date, and follow up status. Tumor size in mm was a continuous variable. Tumor location was classified as upper-inner quadrant (UIQ), upper-outer quadrant (UOQ), lower-inner quadrant (LIQ), lower-outer quadrant (LOQ), nipple/retroareolar, or overlapping. Tumors that were located at 12 o’ clock, 3 o’clock, 6 o’clock, or 9 o’clock based on the clock face method of tumor location were categorized as overlapping. If a patient had multiple tumors located in different quadrants, they were classified as “multicentric” and excluded. If the patient had multiple tumors located in the same quadrant of the same breast, we used the larger tumor as the primary tumor. If the chart documented nipple or retroareolar as the location, we combine-classified the location as nipple/retroareolar. We used primary biopsy to determine the stage, and if the primary tumor was classified as ductal carcinoma in situ-only, the patient was excluded. If the patient’s tumor was classified as ductal carcinoma in situ and another stage, we included the patient. The follow up date was determined by the most recent ambulatory clinic breast notes and follow up status was classified as alive without disease, alive with disease, or deceased. ANOVA test or Student T test was applied to test the association of continuous variables and nodal positivity, and a chi-square test (or Fisher exact test if applicable) was applied to test the association of categorical variables and nodal positivity. All analysis were performed utilizing SAS (Version 9.4, SAS Institute Inc, Cary, NC, USA).

## 3. Results

### 3.1. Demographics

A total of 75 patients were diagnosed with HER2/neu positive primary breast cancer in the selected time frame. Tumors were classified by IHC staining, and Her2 overexpression was confirmed by dual in situ hybridization (DISH), which is the clinical standard at our institution. The mean age at diagnosis was 53.5 years (range, 21–85), and 2.7% of patients were T1mi, 4% were T1a, 12% were T1b, 44% were T1c, 36% were T2, 1.3% were T3. 62.7% were Grade 3. 44/75 (58.7%) of HER2/neu+ patients had nodal metastasis. The mean tumor size was 20.4 mm (range 1–77 mm). Moreover, 75/75 (100%) had SLNB, 37/75patients (49.3%) had ALND, and 72/75 (96%) were clinically node-negative. Over the entire course of this study, clinical evaluation of the axilla was determined by palpation and inspection, and after 2010, routine ultrasound of the axilla was performed on patients with tumors greater than 2 cm on imaging or examination. Table 1 shows the hormone receptor status of the patients involved in this study. The results were: 69.3% were estrogen receptor (ER)+, 62.7% were progesterone receptor (PR)+, and 20% of patients were positive for lymphovascular invasion (LVI). The mean follow up was 65.9 months, with a large range of 0–240 months. Furthermore, 14/75 (18.7%) of patients had a recurrence of their cancer, 7 patients had distance recurrence, 5 had local recurrence, and 2 had locoregional recurrence. Additionally, 90.7% were alive, 9.3% were deceased due to breast cancer, and 73/75 (97.3%) had invasive ductal carcinoma. Only 1/75 (1.3%) had invasive lobular carcinoma, 40/75 (53.3%) had a lumpectomy, and 35/75 (46.7%) had a mastectomy.

### 3.2. Univariate Association with Grade Size and Stage

There was significant association of larger HER2/neu+ tumor size to nodal metastases (*p* ≤ 0.001) and the number of positive lymph nodes (*p* = 0.003). Of note, 21/47 (44.7%) of stage T1 patients had nodal metastasis. Patients with negative nodes had a smaller mean tumor size (*n* = 30; 15.1 mm) than patients with positive nodes (*n* = 45; 23.9 mm) (*p* = 0.002). Lymph node positivity was significantly associated with increasing T-stage (*p* = 0.019). Additionally, the number of positive lymph nodes increased with increasing T-stage, with a cutpoint of stage T2 (*p* = 0.019). Of the 75 patients who had a SLNB, the mean number of positive nodes was 2.4 (range 1–9). Of the 37 patients who had an ALND, the mean number of positive nodes was 1.4 (range 0–15). Lymphovascular invasion was associated with a larger mean tumor size (29.6 mm) than no lymphovascular invasion (20.3 mm; *p* = 0.010). Patients treated with mastectomy had a larger mean tumor size (*n* = 35; 24.5 mm) than patients treated with lumpectomy (*n* = 40; 16.8 mm) (*p* = 0.006). Tumor size was larger in patients who had recurrence of disease, however, it was not statistically significant (*p* = 0.101). T-stage was not associated with recurrence (*p* = 0.160). ER+ and PR+ tumors trended towards larger tumor size, although it was not statistically significant (*p* = 0.303/0.625). There was no significant association between ER+/PR+ status and positive nodes (*p* = 0.117/0.265). 

### 3.3. Sensitivity and Specificity of Imaging

Imaging for nodal disease was performed in 40 patients and findings were suspicious in 36 of them. Axillary ultrasound was the most common modality used; it was performed in 23 patients of whom 14 were found to have pathologically involved nodes. MRI was used in seven patients, and it detected one positive node. Mammograms were performed on nine patients, and three positive nodes were detected. One patient had a mode of imaging classified as “other”, but it did not identify any positive nodes. The sensitivity and specificity of all imaging was 0.50 and 0.77, respectively. Ultrasound had a sensitivity of 0.64 and specificity of 0.67 (Table 2). 

### 3.4. Relationship between Tumor Distance to Nipple and Location to Nodal Status

The mean distance to the nipple measured by ultrasound was 50.84mm (range 0–150 mm). There was a trend in the data showing smaller mean tumor distance to the nipple in patients with positive nodes (*n* = 26; 43.1 mm) versus negative nodes (*n* = 29; 57.8 mm), however it was not statistically significant (*p* = 0.114). Furthermore, 19 patients had a tumor located in the upper-outer quadrant, 19 patients had a tumor categorized as “overlapping”, 11 patients had a tumor located in the lower-inner quadrant, 11 patients had a tumor located in the upper-outer quadrant, 8 patients had a tumor located in the nipple/retroareolar region, and 5 patients had a tumor located in the lower-inner quadrant. There was no significant association between tumor location and nodal disease in this patient cohort. (*p* = 0.861) as shown in Table 2. 

## 4. Discussion

HER2/neu overexpression has been implicated as a negative prognostic factor in patients diagnosed with breast cancer due to higher rates of recurrence and metastasis [3,6,15]. Our study sought to evaluate the relationship between tumor size, location, imaging results and axillary lymph node disease for patients with stage I-III HER2/neu+ breast cancer. Our analysis found that a majority (58.7%) of HER2/neu+ patients had axillary metastasis following surgical excision and SLNB despite being considered clinically node-negative upon physical examination. Our analysis also suggests that tumor size is correlated with increased likelihood of metastasis in HER2/neu+ breast cancer. This is consistent with previous research that larger tumors are more likely to metastasize among all subtypes of breast cancer [2,3,7,15]. 62.7% of HER2/neu+ patients in this study had grade 3 tumors. Increasing tumor grade has been correlated to high probability of axillary lymph node metastasis [2]. Of patients in this study, 20% had LVI, which is another indicator of metastasis in breast cancer [2]. LVI was also significantly associated with larger tumor size. A proposed possible pathogenesis is that Her2/neu overexpression increases angiogenesis by increased VEGF-C production [16,17]. VEGF-C is known to promote lymph node metastasis of tumor cells by increasing lymphangiogenesis in many cancers [15]. Recent study has demonstrated that there is a correlation between VEGF-C overexpression and the increased metastatic risk in breast cancer patients [18]. 

We found that the likelihood of tumor metastasis increased with increasing T-stage, which is consistent with previous studies done on primary breast cancer [4]. However, when compared to previous studies done on all breast cancer subtypes, specific analysis of HER2/neu+ cancer shows a higher rate of metastasis with smaller tumors. Further, 48.6% of HER2/neu+ stage T1 patients had axillary metastasis compared to another study that reported 20.1% of T1 breast cancers had positive axillary lymph nodes [4]. HER2/neu overexpression has been suggested as a predictor of nodal metastasis in small (T1) tumors [7,14]. Understanding the frequency of HER2/neu+ metastasis can help guide surgical management in early stage breast cancer. 

Preoperative AUS was the most frequently used mode of imaging, and ultrasound detected the most suspicious nodes. Suspicious nodes were biopsied under ultrasound. Prior to 2010, AUS was performed based on suspicious clinical exam findings, and after 2010, routine US was performed on tumors 2 cm. Ultrasound is frequently used because it is relatively inexpensive and noninvasive. Our study found that ultrasound had a sensitivity of 0.64 and specificity of 0.67. Other studies have reported values of sensitivity and specificity from 0.61–0.83 and 0.62–0.82, respectively [9,13]. This variability may be explained by one study’s finding that ultrasound is limited when evaluating small ALN or metastasis diameter [9]. Our study had a wide range of tumor sizes, from 1–77 mm, which could affect the accuracy of the ultrasound. One study suggests that imaging modalities should be combined to increase accuracy of the predicted axillary nodal status [9]. The combined sensitivity and specificity of MRI, mammogram, and AUS in our study was 0.50 and 0.77, respectively. These values are not sufficient to suggest forgoing SLNB in the radiologically negative axilla or progressing straight to axillary nodal clearance in the radiologically positive axilla. 

Several studies have proposed that location in the nipple/retro areolar area is a predictor of nodal metastasis in breast cancer [2,4] However, our study did not find a statistically significant relationship between tumor quadrant in the breast and nodal metastasis in HER2/neu+ breast cancer. Tumor distance to nipple has also been reported as an independent predictor of axillary involvement [5]. Our study did not find a statistically significant relationship between smaller tumor-nipple distance and nodal metastasis when we evaluated HER2/neu+ breast cancer. We did see a trend towards smaller tumor distance to the nipple in patients with positive nodes. PR+/ER+ did not predict involvement, which is consistent with findings from a study done on a similar population of surgically treated breast cancer patients [3]. However, several other studies have reported that ER and PR positivity is negatively related to nodal metastasis [7,15]. Further study is indicated in this area to identify the relationship between ER/PR positivity and nodal disease. 

Interestingly, recurrence was not correlated with tumor size or T-stage in this cohort of HER2/neu+ breast cancer. This contradicts current data on recurrence of breast cancer being more likely with larger primary tumor size and higher grade [19,20,21]. This may suggest that factors such as tumor size and stage are not useful predictors of recurrence in HER2/neu+ breast cancer, as it may be more likely to recur regardless of these factors. This is important to consider as an advance in cancer therapy and early detection, meaning that many patients diagnosed with breast cancer will enter remission. The frequency of surveillance of these patients based on risk factors for recurrence such as hormone receptor subtype should continually be evaluated. However, because of the wide range of follow up time in this cohort (.3–240 months), no absolute conclusion on recurrence can be determined by this study. Additionally, our study only included patients treated with upfront surgical resection. Generally, once over 2 cm, HER2/neu+ breast cancer is treated with neoadjuvant chemotherapy and immunotherapy. Therefore, our cohort may have a selection bias towards smaller tumors. There are several reasons why patients over 2 cm may not have received neoadjuvant chemotherapy and therefore were included in our study. First, our study included patients from 2000–2021. This reflects the evolution of care over this time period with the increasing use of neoadjuvant chemotherapy. Furthermore, the decision for neoadjuvant chemotherapy in early stage breast cancer does involve some shared decision-making, and in patients with multiple comorbidities, upfront surgery may have been the recommended or preferred course of therapy. Finally, with the addition routine axillary US after 2010, the population of Her2 patients receiving surgery first was a very selective group, and surgical pathology for these patients may have been recommended due to the potential impact of surgical pathology on adjuvant therapy options.

Our study does have limitations, with the major limitations being the small sample size of 75 HER2/neu+ patients and the inherent selection biases related to changes in clinical practice over time as well as the use of neoadjuvant chemotherapy. In particular, HER2/neu+ patients are often given neoadjuvant chemotherapy, and this may explain the small number found in our query of the database. Analysis with a larger sample size would be needed to determine the relationships reported in this study. Other limitations include the inherent bias and limitations to evaluating long term outcomes that are associated with the retrospective model of data collection.

## 5. Conclusions

Our data suggests that a larger proportion of Her2/neu+ breast cancers have metastatic involvement of axillary lymph nodes. Of note, a large proportion of stage T1 HER2/neu+ tumors had nodal metastasis relative to current rates reported in the literature. Tumor size and stage were the strongest predictors of axillary metastasis. Ultrasound imaging detected the most positive nodes, but did not demonstrate a high sensitivity or specificity. Tumor location, distance to the nipple, and ER/PR positivity did not demonstrate a relationship to nodal disease for HER2/neu+ patients. However, due to the small cohort, further study is warranted.

## Figures and Tables

**Table 1:** Demographics of HER2/neu+ patients.

Variable	Level	N = 75	%
Axillary lymph node dissection	No	38	50.7
	Yes	37	49.3
Grade	1	2	2.7
	2	26	34.7
	3	47	62.7
ER	Negative	23	30.7
	Positive	52	69.3
PR	Negative	28	37.3
	Positive	47	62.7
Alive status	Alive	68	90.7
	Death	7	9.3
Recurrence	No	61	81.3
	Yes	14	18.7
Recurrence Type	Distant	7	50.0
	Local	5	35.7
	Locoregional	2	14.3
SLN *	Yes	75	100.0
Stage T	1mi	2	2.7
	1a	3	4.0
	1b	9	12.0
	1c	33	44.0
	2	27	36.0
	3	1	1.3
Clinical node status	Negative	72	96.0
	Positive	3	4.0
Image type	Mammogram	9	22.5
	MRI	7	17.5
	Other	1	2.5
	US	23	57.5
	Not applicable	35	-
Tumor location	UOQ	19	26.0
	LIQ	5	6.8
	LOQ	11	15.1
	Nipple/Areolar/Retro areolar	8	11.0
	Overlapping	19	26.0
	UIQ	11	15.1
	Missing	2	-
Age at diagnosis	Mean	53.5	
	Minimum	21	
	Maximum	85	
	Std Dev	13.8	
	Missing	0	
Tumor size (mm)	Mean	20.4	
	Minimum	1	
	Maximum	77	
	Std Dev	12.3	
# Of SLN * Positive	Mean	0.8	
	Minimum	0	
	Maximum	4	
	Std Dev	0.8	
	Missing	1	
# of Additional Positive Nodes in ALND	Mean	1.38	
	Minimum	0	
	Maximum	15	
	Std Dev	3.3	
# Of total suspicious nodes in image	Mean	0.53	
	Minimum	0	
	Maximum	4	
	Std Dev	0.9	
Tumor US distance to nipple (mm)	Mean	50.8	
	Minimum	0	
	Maximum	150	
	Std Dev	33.6	
	Missing	20	
Follow-up (months)	Mean	65.93	
	Minimum	0.30	
	Maximum	242	

* Sentinel Lymph Node. Table 1. Showing demographics for the 75-patient cohort examined in this study: 33/75 (44%) of patients were T-stage 1c, and 18.7% of patients had recurrence on follow-up.

**Table 2:** Clinical characteristics associated with nodal disease.

Characteristics	Pathologically Positive Lymph Nodes	Pathologically Negative Lymph Nodes	*p*-Value
Mean Tumor size (mm)	23.9	15.1	0.002
T-stage			0.019
1mi (N = 2)	1	1	
1a (N = 3)	0	3	
1b (N = 9)	3	6	
1c (N = 33)	18	15	
2 (N = 27)	22	5	
3 (N = 1)	1	0	
Image Type			0.027
Mammogram (N = 9)	3	6	
MRI (N = 7)	1	6	
Ultrasound (N = 23)	14	9	
Other (N = 1)	0	1	
Tumor location			0.861
UOQ (N = 19)	11	8	
LIQ (N = 5)	2	3	
LOQ (N = 11)	6	5	
Nipple/Areolar/Retroareolar (N = 8)	6	2	
Overlapping (N = 19)	12	7	
UIQ (N = 11)	6	5	

UOQ: upper-outer quadrant, LIQ: lower-inner quadrant, LOQ: lower-outer quadrant, UIQ: upper-inner quadrant.

## Data Availability

No new data was generated or analyzed in this study. Data sharing is not applicable to this article.
